# Effects of mixed-cooling strategies on executive functions in simulated tennis in hot and humid conditions

**DOI:** 10.3389/fphys.2022.1008710

**Published:** 2022-11-01

**Authors:** Minglang Wen, Guozheng Liu, Wencan Li, Tao Xie, Yukun Zhang, Fei Qin, Jiexiu Zhao

**Affiliations:** ^1^ School of Physical Education, Jinan University, Guangzhou, China; ^2^ School of Economics, Jinan University, Guangzhou, China; ^3^ China Institute of Sport Science, Beijing, China; ^4^ Su Bingtian Center for Speed Research and Training, Jinan University, Guangzhou, China

**Keywords:** hot and humid conditions, heat stress, mixed-cooling strategies, executive functions, tennis

## Abstract

This study aimed to investigate the effects of mixed-cooling strategies, which combines external (cooling vest + neck cooled collar) and internal cooling (cold sports drink ingestion) on measures of executive function during simulated tennis in hot/humid conditions. In a counterbalanced design (randomised order), eight males undertook two trials [one with the mixed-cooling strategy, (MCOOL condition) and another without (CON condition)] in a climate chamber (36.5°C, 50% relative humidity). All subjects completed an intermittent treadmill protocol simulating a three-set tennis match with a 90-second break during odd-numbered games and 120-second breaks between sets, in accordance with the activity profile and International Tennis Federation rules. The mixed-cooling strategies were adopted before test and break time during the simulated tennis match. Stroop task, 2-back task, More-odd shifting task, gastrointestinal temperature (Tgi), skin temperature, blood lactic acid (BLA), heart rate, urine specific gravity (USG), sweat rate (SR), thermal sensation (TS) and perceived exertion (RPE) were measured. Results showed that the mean exercise time was longer in the MCOOL condition than in the CON condition. The SR was greater in CON trial compared with that in MCOOL trial. Results of two-way analysis of variance with repeated measures revealed that time×condition interactions were significant in BLA, Stroop response time, and switch cost of the more-odd shifting task. There were main effects of condition for Tgi, HR, TS, RPE, BLA, Stroop response time, and switch cost of the more-odd shifting task. In a hot/wet environment, pre- and intermittent mixed-cooling strategies can significantly improve exercise time and measures of executive function of tennis players in a simulated tennis match.

## 1 Introduction

High heat stress can reduce physical work capacity and motor-cognitive performances, with consequences for productivity, and increase the risk of occupational health problems (Ebi et al., 2021). Tennis is an intermittently high-intensity sport, and tennis players are usually exposed to heat ambient conditions for training and competition. An epidemiological investigation reported that the Australian Open tennis tournament in Melbourne was disrupted by hot weather, while the core temperatures (Tc) of athletes reached 39°C during competition (Ebi et al., 2021). In addition, related studies indicate that match play in the heat exacerbated the heart rate and perceived exertion, and worsen the hydration status and thermal comfort (Bergeron, 2014). Moreover, maximal voluntary strength in the lower limbs and repeated-sprint ability deteriorates in the heat (Girard et al., 2014; Périard et al., 2014). Therefore, hot ambient conditions have become a problem when tennis players train and compete.

Exercise at an extremely high ambient temperature caused hyperthermia (Tc > 38.5°C) (Mora-Rodriguez, 2012), and a high level of hyperthermia impairs cognitive function (Schmit et al., 2017). Tennis is a multifactorial sport; it not only needs a combination of physical fitness such as speed, strength, agility, and aerobic endurance, but also mental capacity including anticipation, reaction and executive function (Hornery et al., 2007). In the field of exercise performance, executive functioning refers to the ability to remain lucid and solve problems during the tough stages of exercise (Tsai et al., 2017). Specifically, tennis players are required to initiate immediate adjusting behaviours in response to a direct opponent, alter a pacing strategy, inhibit an impulsive response, or distinguish between unfair and fair decision. Common executive functions include inhibitory control, working memory and cognitive flexibility (Diamond, 2013). Inhibitory control is the ability to override strong internal predispositions or external distractions. Working memory is the ability to keep information in mind and manipulate it. Cognitive flexibility involves thinking about a particular subject in multiple ways, quickly switching between tasks and shifting attention. Moreover, a significantly lower colour-word score after 60 min running of collegiate male athletes (soccer, *n* = 5; rugby, *n* = 5) indicated the worse executive functions in heat conditions (Roh et al., 2017). However, the research examining the effects of high ambient temperature on executive functions for tennis players are limited.

Effective cooling strategies alleviate the adverse effects of heat conditions and improve exercise performance and capacity of athletes in extreme heat environments (Schranner et al., 2017; Lynch et al., 2018; Gibson et al., 2020). Cooling strategies have attracted special attention from the human science community. The type of cooling strategy, cooled body area and duration of application of the cooling strategies vary between sports (Ranalli et al., 2010). The optimal cooling strategy is dependent on the types and characteristic of sports. For example, applying cooling measures at intervals maintains lower thermal stress throughout the duration of the exercise (Douzi et al., 2019). Unlike in other sports, application of cooling interventions pre-game, between game breaks and during set breaks in tennis matches are necessary (Schranner et al., 2017). In recent years, most published studies were focused on the physiological effect of cooling using the above-mentioned interventions during breaks in tennis matches in heat environments (Schranner et al., 2017; Lynch et al., 2018). The study also found that crushed ice ingestion and mid-cooling by menthol swilling lessened the performance decline in cognitive function when long-distance runners were running in hot and humid conditions (Saldaris et al., 2020). However, the effect of intermittent cooling strategies on the potential impariments of executive functions induced by heat stress of tennis players is unknown.

Therefore, the main purpose of this study was to evaluate the effect of intermittent mixed-cooling strategies on the measures of executive functions (inhibitory control, working memory, and cognitive flexibility) of tennis players in heated conditions. The study hypothesised that intermittent mixed-cooling strategies will improve the executive functions of tennis players in a hot environment. The findings from this research may provide a relevant theoretical and experimental basis strategy to maintain an effective psychological state in heat environments.

## 2 Materials and methods

### 2.1 Participants

Eight collegiate male tennis athletes (age: 21.75 ± 2.2 years, height: 174.9 ± 4.8 cm, weight: 67.1 ± 7.5 kg, training years: 6.9 ± 2.6 years), non-acclimatised to heat, volunteered to participate in the study. Participants did not have any sports injuries in the last 6 months, or any hereditary and chronic diseases. Participants were instructed to avoid caffeine or alcohol in the 12 h before each trial and to avoid strenuous exercise in the 48 h before each trial. All participants signed informed consent before the experiment. All experimental procedures were approved by the Jinan University Human Research Ethics Committee (JNUKY-2020-010).

### 2.2 Overall design

Participants attended two different trials in a counterbalanced order. The control trial was a simulated match-play tennis in high ambient temperature without any cooling interventions (CON). The mixed-cooling intervention trial (MCOOL), which combines external (cooling vest + neck cooled collar) and internal cooling (cold sports drink ingestion), was a simulated match-play tennis in high ambient temperature with pre-cooling and in-play cooling interventions. The time of testing was kept constant within each subject, and separated by 7 days. All trials were completed in a climate-controlled chamber in the Special Environment Laboratory at the Anta Co., Ltd. (Guangzhou, China). The climate chamber was regulated at an air temperature of 36.3–36.8°C and controlled humidity of 45%–55%.

After 10 min of mixed-cooling, participants immediately warmed up on the treadmill with a self-chosen intensity for 5 min, which was the same between all trials. After the warm-up, they were ready to start a simulated tennis match. Participants were treated with the mixed-cooling strategy during the odd game rest and set rest periods. Heart rate was monitored in real-time during the exercise (920XT, GARMIN, United States), as well as gastrointestinal temperature. Thermal sensations and rating of perceived exertion (RPE) were collected during the rest period. Skin temperature (Tsk), urinary specific gravity, body weight, blood lactic acid (BLA) and executive function tests (Stroop; 2-back; More-odd shifting paradigm) were collected before precooling and after the simulated tennis match.

### 2.3 Simulated tennis match protocol

The exercise programme simulated the metabolic load of a tennis match, referred to Schranner and Lynch’s exercise protocol (Schranner et al., 2017; Lynch et al., 2018) with a modified exercise load. Before the experiment, we used a portable ergospirometer and a heart rate monitor to measure the exercise intensity and load of 1 point in the actual tennis match of college tennis athletes. VO_2max_ was measured to set exercise intensity before 3 days of the test. The exercise intensity and load of the experimental protocol were comparable to the actual tennis match of college tennis athletes (%VO_2_max: 50%–60%) by regulating the speed of treadmill. To simulate one “point”, the participant ran at 14 km·h^−1^ for 9–11 s (including 3 s of acceleration time), and then jogged at 6 km·h^-1^ speed for 19–21 s (including 3 s of deceleration time). The total duration of one “point” is 30 s, six “points” constituted one game, nine “games” constituted one “set” and three “sets” were completed in each trial. Trials were terminated early if the gastrointestinal temperature (Tgi) exceeded 39.5°C or volitional exhaustion occurred.

### 2.4 Mixed-cooling strategies

**FIGURE 1:**
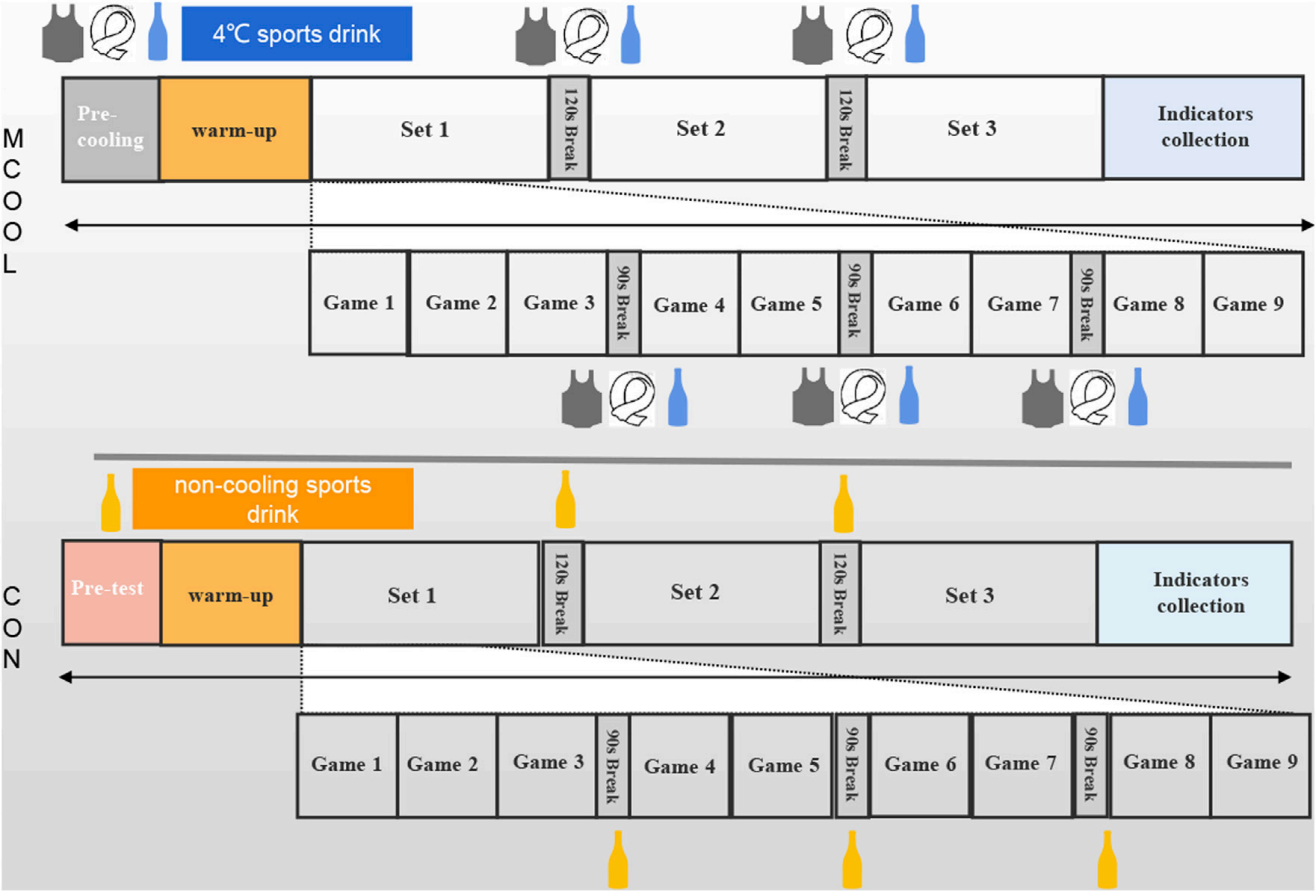
Mixed-cooling strategies.

For the MCOOL condition, participants underwent a 10-minute pre-cooling intervention (cooling vest + neck cooled collar +4°C cold sports drink ingestion). According to tennis rules, a 90-second break is allowed after the end of an odd game, and a 120-second break is allowed after a player has won six games. Mixed-cooling strategies were adopted in the above break time during the simulated tennis match. By contrast, the control trial was only allowed to ingest the non-cooling sports drink (23°C) during the pre-cooling intervention and the same rest time in the simulated tennis games. The same volume of sports drink was supplied in CON and MCOOL trial. The amount of sports drink ingested was recorded in both trials. Figure 1 shows the details of the mixed-cooling strategies.

### 2.5 Measurements

#### 2.5.1 Measurement of executive functions

##### 2.5.1.1 Stroop task

The Stroop paradigm was used to evaluate the inhibition control level of the athletes. All Stroop task were tested in PsyToolkit’s Experiment Library (https://www.psytoolkit.org/experiment-library/). English words with colours (such as red, green, yellow and blue) will appear in the Stroop test, but these English words’ font colour and meaning may not be the same. For example, the word “BLUE” may be in green font colour. The subjects were asked to determine the colour of the word and press the corresponding button (“R” for red, “G” for green, “Y” for yellow and “B” for blue). The test lasted no more than 2 minutes and featured 40 trials. In this experiment, the Stroop effect was used to represent the performance of this task, that is, the difference between inconsistent test responses and consistent test responses. All participants completed 3 times practices before 1 hour of the actual trial to ensure that the Stroop task was sufficiently understood. The data of Stroop task were collected one time before precooling and after the simulated tennis match.

##### 2.5.1.2 2-Back task

The 2-back paradigm was used to evaluate the working memory. All 2-back task were tested in PsyToolkit’s Experiment Library (https://www.psytoolkit.org/experiment-library/). The 2-back task consisted of three blocks (25 trials each). The stimuli were arranged in a pseudo-randomised order. All runs were matched for the letter of targets (33%) and non-targets (67%), as well as for distractors (e.g., 2-back targets in a 3-back run). The total stimulus set is 15 stimuli (letters), and each letter will appear for 500 milliseconds. After 500 milliseconds, a black screen will appear for 3,000 milliseconds for subjects to judge whether the letters that appear this time are consistent with the first two letters. If yes, the participants should press “M,” and they will see a green shade around the letters. If participants pressed the wrong button, then they will see a red shade around the letter. If inconsistent, then no response is required until the first two are consistent. In this test, the accuracy rate and reaction time represents the achievement of the task. All participants completed 3 times practices before 1 hour of the actual trial to ensure that the 2-back task was sufficiently understood. The data of 2-back task were collected one time before precooling and after the simulated tennis match respectively.

##### 2.5.1.3 More-odd shifting task

The more-odd shifting task paradigm is a test used to assess cognitive flexibility. All task were conducted in PsyToolkit’s Experiment Library (https://www.psytoolkit.org/experiment-library/). More-odd shifting is a consonant-vowel-parity shifting task. The test was divided into three types of blocks (40 trials each). In the Block A, only the upper part of the screen was stimulated, and the subjects were asked to determine whether the word part was a vowel (N, not B for vowels). In the Block B, only the lower part of the screen was stimulated, and the subjects were asked to judge whether the number part was odd (press “B” for odd numbers, not “N” for odd numbers). In the Block C, four squares on the screen are stimulated clockwise. We assessed the switch cost of the more-odd shifting task, which was defined as the difference of RTs (response time) between the switching trials (i.e., block C) and the non-switching trials (i.e., block A and B). All participants completed 3 times practices before 1 hour of the actual trial to ensure that the more-odd shifting task was sufficiently understood. The data of more-odd shifting task were collected one time before precooling and after the simulated tennis match respectively.

#### 2.5.2 Measurement of temperature regulation

Core temperature was measured using the VitalSenses telemetric physiological monitoring system (Mini Mitter Co. Inc., Bend, Oregon, United States), which includes a receiver and a thermal-based ingestible temperature sensor capsule to transmit Tgi. Four hours before each session, subjects swallowed a temperature sensor capsule to ensure that it had passed into the gastrointestinal tract and would be insensitive to temperature changes resulting from fluid intake during testing.

The pre and post Tsk of participants were measured before and after the experiment using a thermocouple thermometer (YHT309, Shenzhen Yuan Heng Tong Technology, China) at the following locations: chest temperature (T_chest_), arm temperature (T_arm_), thigh temperature (T_thigh_) and forehead temperature (T_forehead_). Average skin temperature is calculated as follows: $Tskin=\left(0.1\times T_{forehead}\right)+\left(0.6\times T_{chest}\right)+\left(0.2\times T_{thigh}\right)+\left(0.1\times T_{arm}\right)$ (Wilmore, 1994).

#### 2.5.3 Hydration status and blood lactic acid

Urine samples were collected before and after the trial to detect the urine specific gravity using the digital handheld urine specific gravity refractometer (PAL-10S, ATAGO, Japan). In addition, nude body mass was measured using a digital weighing scale (AH100, Huawei, China; precision of 0.02 kg) before and after the trial, which was corrected for fluid ingestion, urine excretion, and blood removal to estimate sweat rate $\left(L\cdot h^{-1}\right)$ *via* the following equation (Stevens et al., 2017b):

Sweat rate=(Δbody mass+fluid−urine−blood)/exercise time

BLA was measured before and after the experiment to compare subjects’ exercise intensity and fatigue degree after exercise in a high-temperature environment. Blood was collected from a fingertip prick to determine BLA concentration using a Lactate Scout portable lactate test analyser (EKF Diagnostics, Berlin, Germany).

#### 2.5.4 Perceptual data

Thermal sensation (TS) was measured using a thermal sense scale to monitor the heat tolerance of subjects in real time. Ten scales ranged from 0 (very cold) to 9 (very hot). The RPE was measured using a modified Borg scale, with standard increments from 6 (no effort at all) to 20 (exhausted). Subjects’ TS and RPE were obtained before the experiment and during the rest of each session.

### 2.6 Statistical analysis

All data were detected for normality through the Shapiro–Wilk test. Paired sample *t* test was used to compare the parameter in each condition (exercise time and sweat rate). Two-way repeated measures ANOVA (Condition: CON and MCOOL; time: set1, set2 and set3) was used to analyse Tgi, △ HR, TS and RPE. Also, two-way repeated measures ANOVA (Condition: CON and MCOOL; time: pre- and post-) was used to analyse Stroop response time, 2-back correct rate, 2-back reaction time, more-odd switch cost, Tsk, BLA, and urine specific gravity. When significant interaction effect of condition by time was found, simple effect of condition and time was analysed respectively using Bonferroni’s post hoc test. When the interaction effect of condition by time is not significant, only the main effect of time and condition are analysed. *p* < 0.05 was taken as the level of significant difference. All statistical analyses were performed using SPSS (Version 23.0; IBM SPSS Inc., Chicago, IL). Results are reported as mean ± standard deviation. In addition, effect size estimates (Cohen’s d) were calculated to assess and categorise efficacy as small (*d* = 0.2), medium (*d* = 0.5), or large (*d* = 0.8) (Lakens, 2013).

## 3 Results

### 3.1 Exercise time/heart rate

**FIGURE 2:**
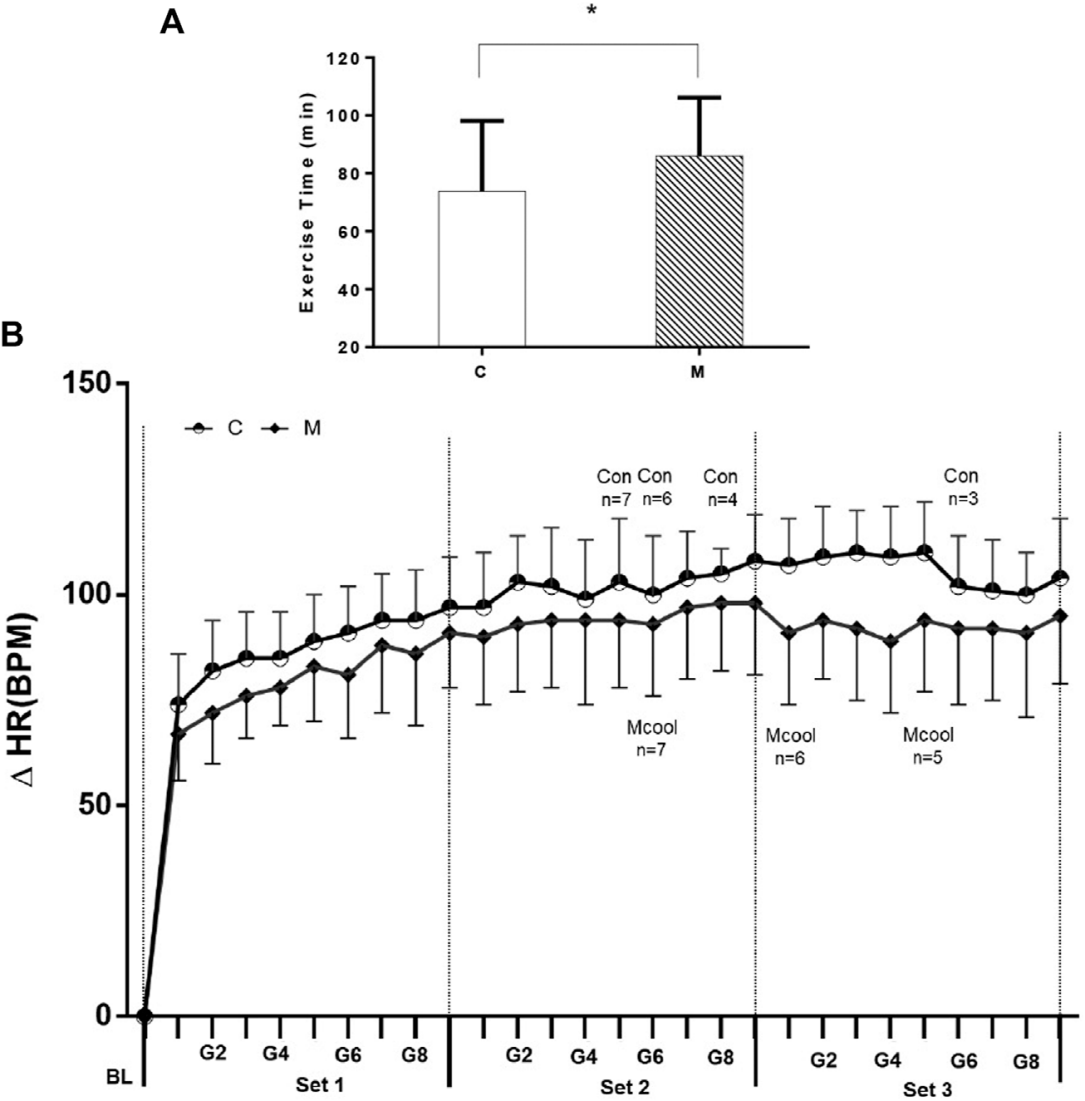
**(A)** Mean exercise time of simulated tennis match in both CON and MCOOL conditions. **(B)** Changes in heart rate (HR) over the entire simulated tennis match between CON and MCOOL groups. Vertical lines in **(B)** denote the end of each “set”. **(B)** indicates the remaining participants (*n*) out of eight for each trial. * Indicates *p* < 0.05 between groups. All values are presented as Mean ± SD. M, mixed-cooling condition; C control condition.

A total of eight participants participated in the simulated tennis match in both CON and MCOOL conditions. Three participants completed the CON trial, and the reasons for withdrawal are exhaustion (*n* = 3) or reaching the termination core temperature criterion (Tgi >39.5°C, *n* = 2). Five participants completed the MCOOL trial, and the participant withdrawals because of exhaustion (*n* = 2) or reaching the termination core temperature criterion (*n* = 1). The mean exercise time was longer (CON: 73.81 ± 24.30 vs. MCOOL: 86.06 ± 20.08 min; *p* = 0.043, Cohen’s *d* = 0.55) in the MCOOL condition (Figure 2A) than in the CON condition.

Figure 2B shows the changes in heart rate throughout the simulated matches. No interaction of △HR was found throughout the simulated tennis match (condition × time, F = 0.187, *p* = 0.831). However, there was a condition main effect (F = 5.289, *p* = 0.029), which suggested that Mixed-cooling strategy can reduce HR during exercise in heat condition. Additionally, there was a time main effect (F = 4.510, *p* = 0.020), in which △HR was rising visibly during exercise (Figure 2B).

### 3.2 Thermoregulatory responses

**FIGURE 3:**
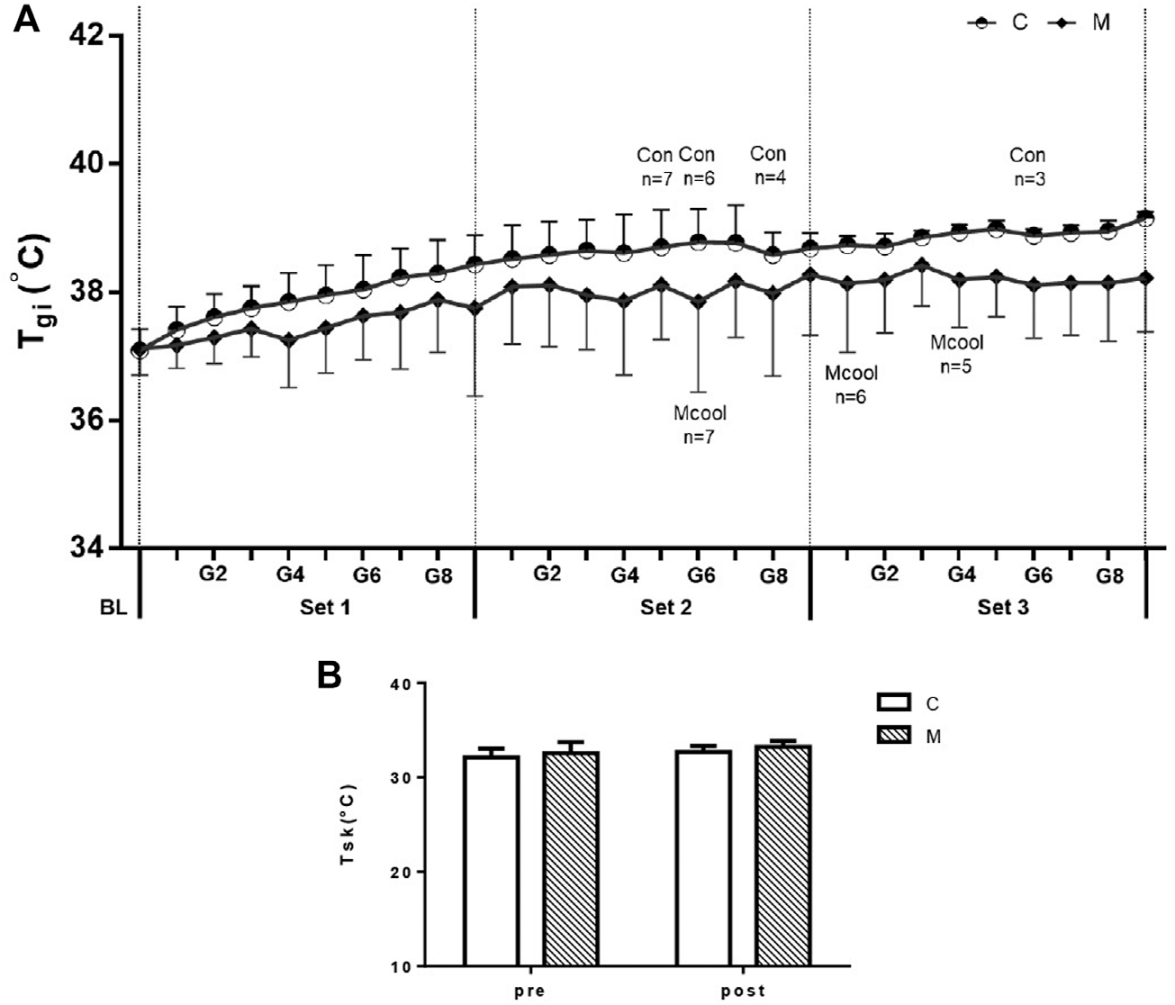
Mean T_gi_ values **(A)** and mean T_sk_ values **(B)** in the simulated tennis match between CON and MCOOL conditions. Vertical lines in **(A)** denote the end of each “set”. **(A)** indicates the remaining participants (*n*) out of eight for each trial. All values are presented as Mean ± SD. M, mixed-cooling condition; C, control condition.

Gastrointestinal temperature and skin temperature were collected to evaluate thermoregulatory responses, as shown in Figure 3. No interaction of Tgi was observed during the simulated tennis match (condition × time, F = 0.186, *p* = 0.831). However, there was a condition main effect (F = 6.396, *p* = 0.017), which indicates that Mixed-cooling strategy can reduce Tgi during exercise in heat condition. Moreover, Tgi had a main effect for time (F = 4.289, *p* = 0.023), thereby reflecting that Tgi gradually rose during the entire simulated tennis match (Figure 3A). However, no interaction (condition × time, F = 0.038, *p* = 0.850), condition main effect (F = 3.156, *p* = 0.119) and time main effect (F = 2.272, *p* = 0.175) of Tsk (Figure 3B) were observed during the simulated tennis match.

### 3.3 Hydration status

**FIGURE 4:**
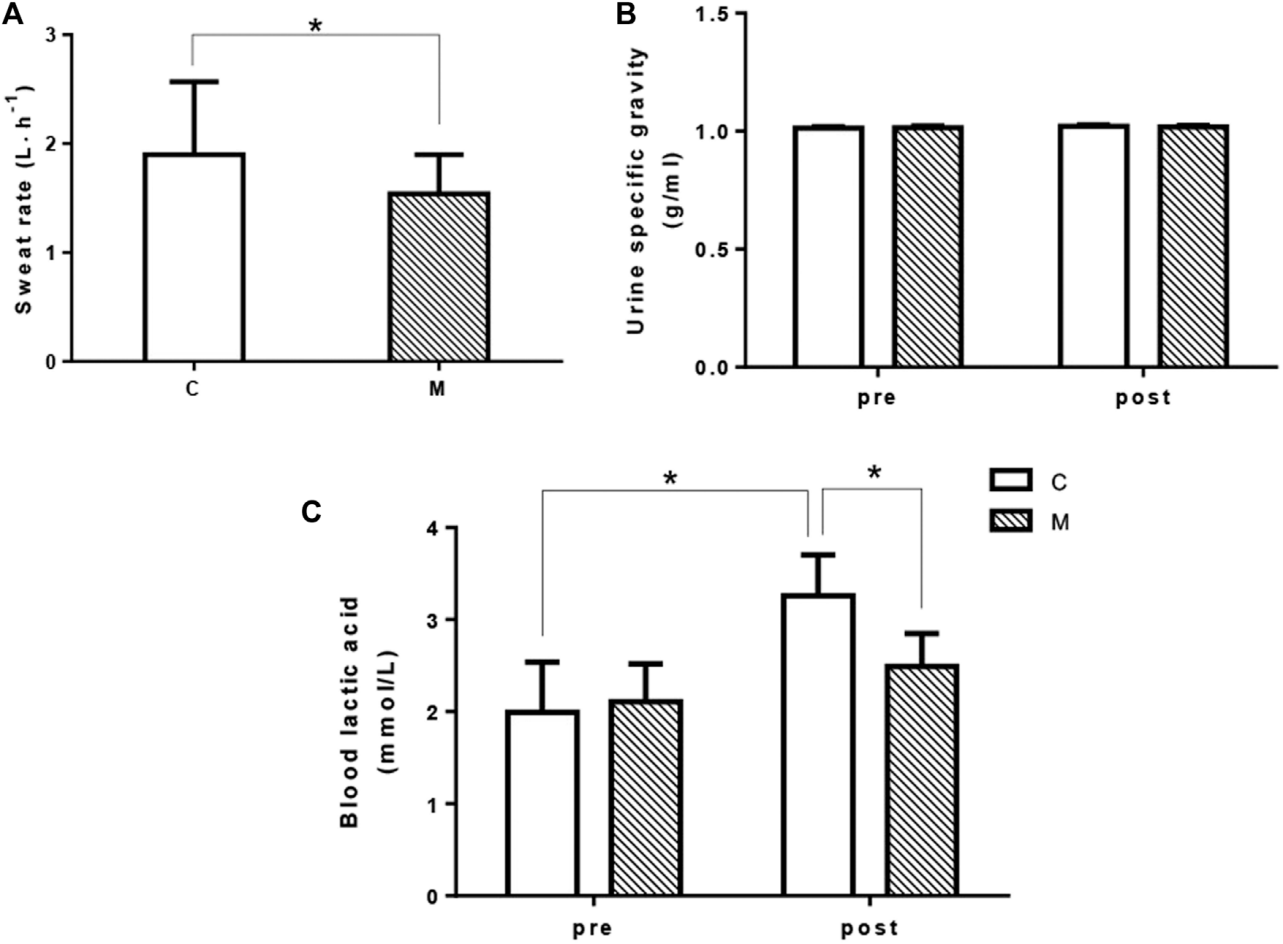
Changes in sweat rate **(A)**, urine specific gravity **(B)**, and blood lactic acid **(C)** in the simulated tennis match between CON and MCOOL conditions. *Indicates *p* < 0.05 between groups. All values are presented as Mean ± SD. M, mixed-cooling condition; C, control condition.

Hydration status was evaluated by using urine specific gravity and sweat rate as shown in Figure 4. The sweat rate was greater in CON trial compared with that in MCOOL trial (*p* = 0.049, Cohen’s d = 0.669, Figure 4A). Moreover, no interaction (condition × time, F = 0.765, *p* = 0.411) and condition main effect (F = 0.101, *p* = 0.759) of USG was observed during the simulated tennis match (Figure 4B). However, USG had a main effect for time (F = 8.481, *p* = 0.023), thereby reflecting that USG gradually rose during the simulated tennis match. These results reflected that mixed-cooling intervention minimized the extent of sweat rate during exercise in heat condition.

### 3.4 Blood lactic acid levels

Blood lactate level is a common marker of physiological response to exertion. A significantly interaction of BLA was found during the simulated tennis match (condition × time, F = 18.439, *p* = 0.004). There was a condition main effect (F = 5.633, *p* = 0.049). The BLA level was obviously higher in CON condition after exercise compared with that of in MCOOL condition (*p* = 0.005, Cohen’s *d* = 1.915). Moreover, BLA had a main effect for time (F = 28.658, *p* = 0.001), which indicating that BLA was gradually enhanced during the entire simulated tennis match (Figure 4C).

### 3.5 Perceptual data

**FIGURE 5:**
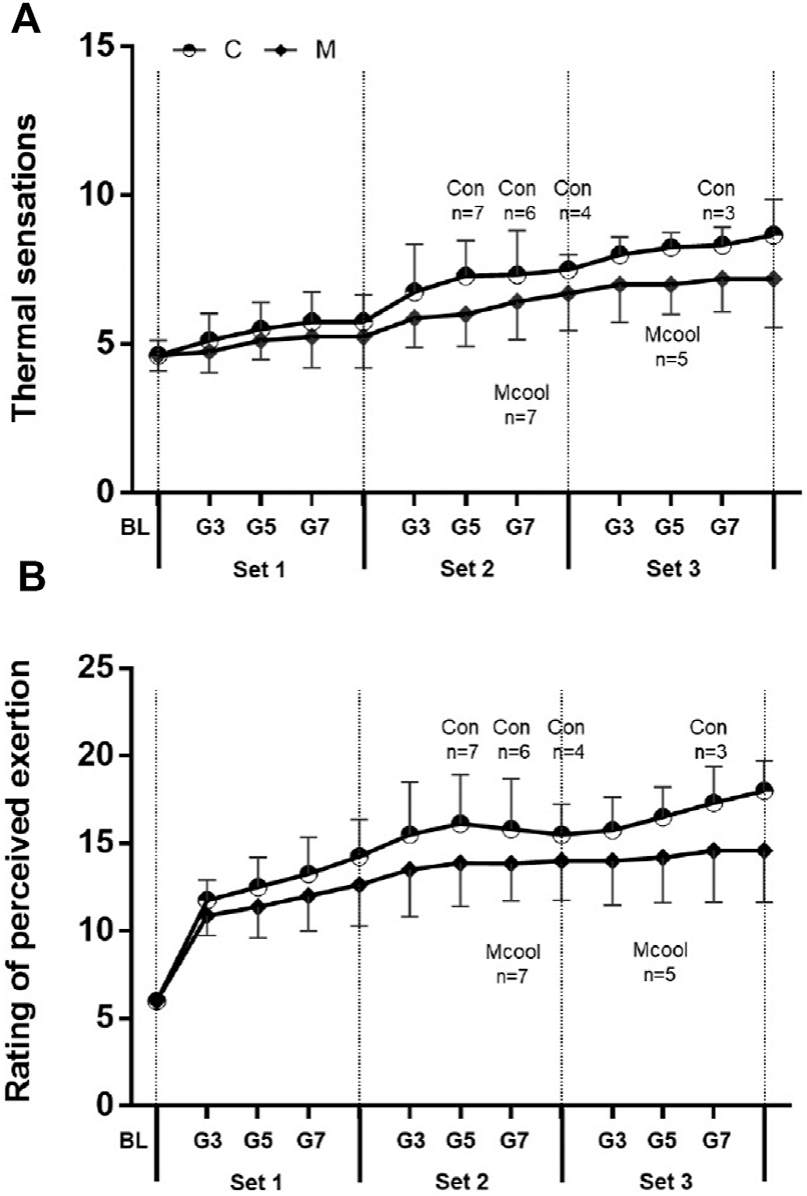
Thermal sensations **(A)** and rating of perceived exertion **(B)** over the entire simulated tennis match in CON and MCOOL conditions. Vertical lines denote the end of each “set”. Remaining participants (*n*) out of eight are indicated for each trial. All values are presented as Mean ± SD. M, mixed-cooling condition; C, control condition.

No interaction of TS was identified during the simulated tennis match (condition × time, F = 0.303, *p* = 0.741). However, there was a condition main effect (F = 4.503, *p* = 0.043), indicating that Mixed-cooling strategy can reduce TS during exercise in heat condition. Moreover, TS had a main effect for time (F = 13.387, *p* = 0.000), indicating that TS was gradually enhanced during the entire simulated tennis match (Figure 5A). Figure 5B presents the exercise perception during the simulated match-play activity. No interaction of RPE was found during the simulated tennis match (condition × time, F = 0.381, *p* = 0.687). There was a condition main effect (F = 4.237, *p* = 0.049), indicating that Mixed-cooling strategy can reduce RPE during exercise in heat condition. In addition, RPE had a main effect for time (F = 6.749, *p* = 0.004), indicating that RPE was gradually enhanced during the entire simulated tennis match.

### 3.6 Executive function

**FIGURE 6:**
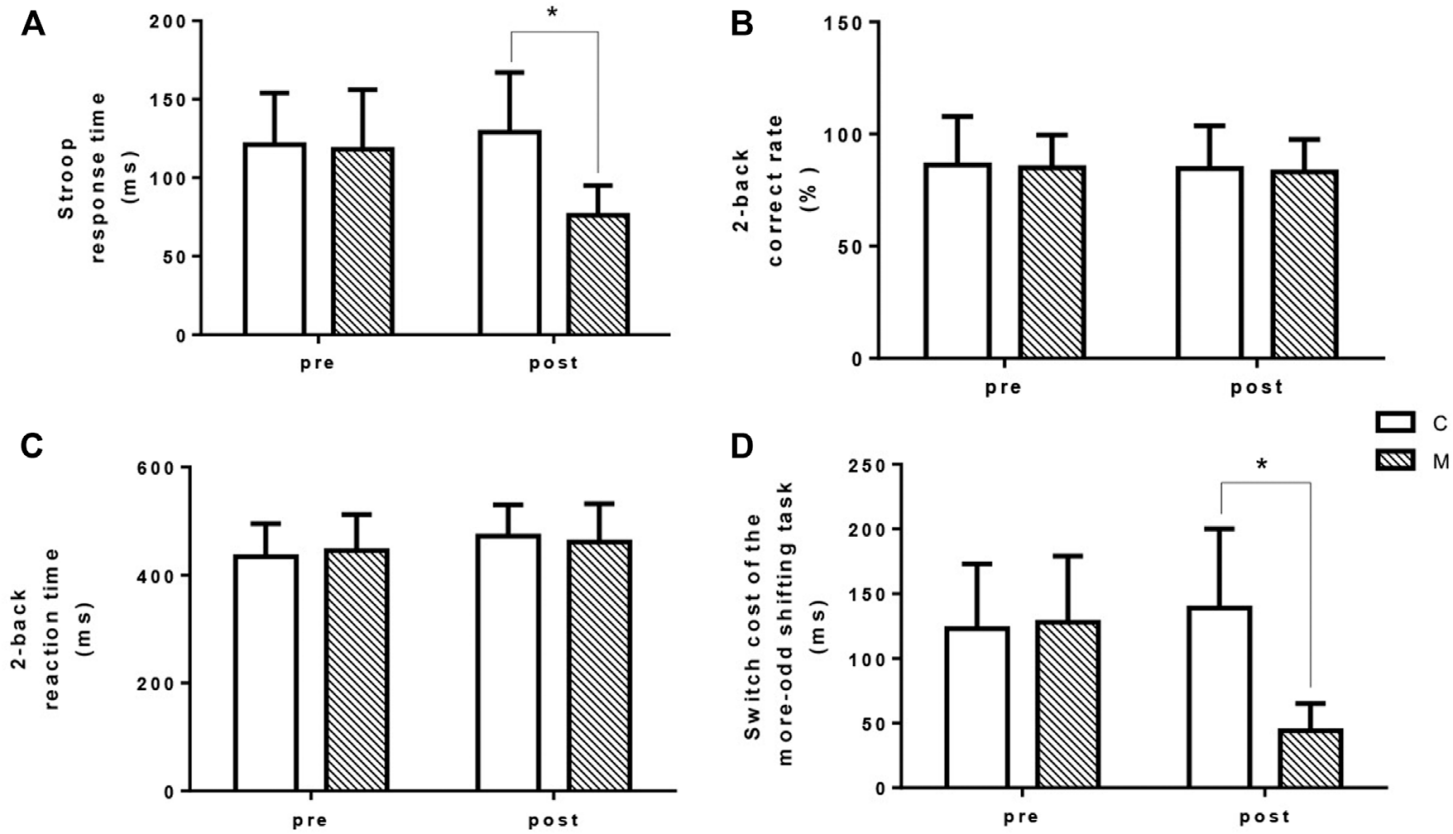
Changes in Stroop response time **(A)**, 2-back correct rate **(B)**, 2-back reaction time **(C)**, and switch cost of the more-odd shifting task **(D)** pre- and post-simulated tennis match in both CON and MCOOL conditions. *Indicates *p* < 0.05 between groups. All values are presented as Mean ± SD. M, mixed-cooling condition; C, control condition.

As a marker of response inhibition, Stroop test was performed prior to and after the CON and MCOOL trials (Figure 6A). A significantly interaction of Stroop response time was found during the simulated tennis match (condition × time, F = 23.291, *p* = 0.002). There was a condition main effect (F = 9.159, *p* = 0.019), in which the Stroop response time was obviously higher in CON condition at post-exercise compared with that of in MCOOL condition (*p* = 0.001, Cohen’s *d* = 1.764). Moreover, there was no main effect for time (F = 1.734, *p* = 0.229) of Stroop response time during the entire simulated tennis match. N-back (2-back) paradigm was performed to assess the working memory of participants (Figures 6B,C). No interaction (condition × time, F = 0.001, *p* = 0.982), condition main effect (F = 0.025, *p* = 0.879), and time main effect (F = 0.426, *p* = 0.535) of 2-back correct rate was found during the simulated tennis match (Figure 6B). Meanwhile, no interaction (condition × time, F = 0.641, *p* = 0.450), condition main effect (F = 0.000, *p* = 0.990), and time main effect (F = 1.706, *p* = 0.233) of 2-back reaction time was also observed during the simulated tennis match (Figure 6C). More-odd shifting task paradigm was performed to assess cognitive flexibility (Figure 6D). A significantly interaction of switch cost of the more-odd shifting task response time was found during the simulated tennis match (condition × time, F = 23.291, *p* = 0.002). There was a condition main effect (F = 9.159, *p* = 0.019), in which the switch cost time was obviously higher in CON condition post exercise compared with that of in MCOOL condition (*p* = 0.009, Cohen’s *d* = 2.082). Moreover, there was no main effect for time (F = 1.734, *p* = 0.229) of switch cost during the entire simulated tennis match. These results indicate that mixed-cooling intervention improve the executive function in high temperature humidity environment, especially response inhibition and cognitive flexibility.

## 4 Discussion

This study assesses the efficacy of pre-cooling and intermittent mixed-cooling strategies applied during mandated breaks in the simulated tennis match-play activity on mitigating executive functions (inhibitory control, working memory and cognitive flexibility) in hot/humid conditions. These findings specifically reinforce that pre-cooling and intermittent mixed-cooling strategies can significantly improve exercise time and executive function during a simulated tennis match in high-temperature and humid environments. The improvements in executive function are particularly evident in the areas of inhibitory control and cognitive flexibility. Elevated body temperature and dehydration are potential factors that may affect the executive function and exercise performance of athletes. The findings from this research may provide a relevant theoretical and experimental basis strategy to maintain an effective psychological state of tennis players in heat environments.

Environmental conditions of 36°C, 50% RH were the typical conditions for the US Open and Australian Open in recent years (Schranner et al., 2017). Above environmental parameters are similar the relative studies (Bergeron, 2014; Schranner et al., 2017), which can be simulated heat condition in tennis tournaments. Under the very hot/humid ambient conditions in our study, five of the eight participants could not complete the three-set simulated tennis match because of exhaustion or reaching the termination core temperature criterion without the mixed-cooling strategies. Even with the cooling strategy, three participants stopped before the end of the trial. Such numbers confirms that the heat climate caused great heat stress to the participants. Based on the characteristics of the tennis formats and exercise intensity, the three-set simulated tennis match was adopted. The exercise protocol combined bouts of short-duration fast running and intermediate periods of walking (Schranner et al., 2017; Lynch et al., 2018). Participants were required to run at the same intensity in all sets, which was necessary to isolate the independent influence of cooling strategy on thermal strain. Furthermore, the total duration of one “point” is 30 s, six “points” constituted one game, nine “games” constituted one “set” and three “sets” were completed in each trial. In this way, the situation simulated a tough scenario, which posed further challenge to the tennis players.

In this study, we selected the mixed-cooling strategy, which combines external (cooling vest + neck cooled collar) and internal cooling (cold sports drink ingestion) to improve executive functions and exercise time of tennis players in hot/humid conditions for the following reasons. Firstly, in order to obtain optimum effect of cooling, we chose external cooling techniques to lower the skin and core temperature in the heat (James et al., 2015). Additionally, internal cooling (cold sports drink ingestion) was used as a gastrointestinal heat sink to reduce the central body temperature, increase the capacity for heat storage (Jay and Morris, 2018) and prevent dehydration. Secondly, the torso (cooling vest) was presented as an effective cooled area due to its large vascularisation and numerous cold receptors, thereby cooling substantial quantities of blood (Stevens et al., 2017a; Xu et al., 2021). Moreover, cooling the neck (neck cooled collar) is strongly recommended during mandated breaks in tennis match-play because the neck is in closed proximity to the thermoregulatory centre and is an area of high alliesthesial thermos sensitivity (Tyler and Sunderland, 2011a; Tyler and Sunderland, 2011b). Thirdly, this study combines the characteristics of tennis format and the feasibility of cooling strategies. One of the strategies is pre-cooling, which reduced the body temperature before exercise to enhance the capacity of heat storage (Jones et al., 2012; Wegmann et al., 2012). However, the beneficial effects of pre-cooling are often lost during exercise. Hence, intermittent cooling was used during mandated breaks to achieve sustained cooling throughout the tennis tournament. Overall, the benefits of the cooling strategy on exercise time and executive function were proven in our research.

Higher-level cognitive functions such as executive functions are involved in the control and regulation of “lower-level” cognitive processes such as reaction time, and reflect the use of strategies that are necessary for solving a problem and setting goals (Alvarez and Emory, 2006; Diamond, 2013). Several studies have proven that executive functions, including inhibitory control, working memory and cognitive flexibility, are important for performance in open-team sports (Huijgen et al., 2015; Elferink-Gemser et al., 2018). Open-team sports involve interaction and estimation with direct opponents and as such lead to situations with numerous unstable and unpredictable events (Singer, 2000). Competitive tennis players are exposed to such situations on a regular basis in their training and when competing. Our research used the Stroop test, 2-back test and more-odd shifting tests to evaluate inhibitory control, working memory and cognitive flexibility, respectively. Meta-analysis found that there were immediate improvements in executive function specifically after aerobic exercise (Chang et al., 2012). This study found that the improvements induced by exercise in executive function were not observed in high temperature and humidity condition (36°C, 50%RH). Similarly, Shibasaki et al. (2019) found that executive functions decreased after moderate-intensity exercise at heat condition (35°C, 30%–40% RH) (Shibasaki et al., 2019). Notably, significant improvements were found in Stroop response and more-odd shifting test after using pre-cooling and intermittent mixed-cooling strategies, which are inconsistent with the findings of Tessa Maroni (Maroni et al., 2018) and Jyh-How Huang (Huang et al., 2022). Compared with the cooling interventions in the above two studies, the cooling interventions in our study had three main differences. Firstly, Tessa Maroni and Jyh-How Huang did not conduct pre-cooling. Secondly, according to tennis rules, our cooling strategy has more times cooling intervention (cooling intervened in 90-second and 120-second break time, up to twelve times cooling intervention during the game) compared with that of Jyh-How Huang (three to four times cooling intervention during the game). Lastly, the above studies did not apply internal cooling procedures. The above differences in cooling effect further suggest that the duration, density and mode of cooling will affect the intervention effect of athletes’ executive function in heat. Pre-cooling and intermittent mixed-cooling strategies used in our research can improve inhibitory control and cognitive flexibility in simulated tennis match-play activities in a high-temperature and humid environment. However, this study found that changes in working memory were not observed even after using mixed-cooling measurement. This result was consistent with those of Toru Ishihara (Ishihara and Mizuno, 2018) on the cognitive ability of juvenile tennis players. Exercise frequency and competition experience were more important factors than cooling intervention affecting working memory (Schranner et al., 2017). The result also suggests that the mixed-cooling measures and heat condition have little effect on the working memory ability of the subjects.

When exercise in hot condition, skin blood flow raise in order to lower body temperature, which will lead to a relative decrease in the brain blood supply compared with that of exercise in normal temperature (Nybo et al., 2002; Nielsen and Nybo, 2003). Moreover, the increased temperature in brain worsens the function of nerve cells (Nielsen and Nybo, 2003). A decline in core temperature (Tgi) was evident throughout the entire simulated tennis match with the strategies. These improvements of core temperature in hot/humid conditions with mixed-cooling strategies are consistent with those of published research (Lynch et al., 2018) (Naito et al., 2022). One of the primary objectives of cooling interventions was to reduce core temperature and brain temperature, leading to increased capacity for heat storage. The decreased temperature of the blood flowing through digestive system can reduce the temperature of cerebral blood, then reduced thermal sensation and the stimulation of thermoreceptors which influence areas of central drive in the brain (Maughan et al., 2007). Additionally, the Mixed-cooling strategies would increase the blood supply and nerve cell function of the brain, further alleviated metabolic and circulatory perturbations within the brain (Maughan et al., 2007). Core temperature may be a one of factors that is responsible for worse in executive functions in heat conditions. In the early stages of simulated tennis implied that pre-cooling may act as a thermal buffer, delaying the higher core temperature and deleterious effects of exercise in the heat on cognitive function. Moreover, intermittent cooling may serve as a cooling battery, alleviating the rising trend of core temperature in the latter stages of the tennis tournament. However, dehydration is one of the potential factors affecting the executive function of athletes in high-temperature and humid environments. Heat stress-induced hydration has adverse effects on executive function (Roh et al., 2017). Our study proved that the mixed-cooling measures can effectively maintain athletes’ hydration state by reducing sweat loss. Our research data also suggested that compared with ingesting lukewarm sports drink, cold sports drink ingestion probably not only compensated water loss timely, but also declined the core temperature, thereby reducing sweat rate and maintaining a premium hydration state.

To ensure strict environmental conditions, the present study used a treadmill in a climate chamber to simulate specific tennis activities. Such an approach could be a limitation. However, the intermittent activity profile of the sport and exercise intensity were replicated and the metabolic heat production produced by the exercise protocol was the same as previously reported during live tennis match-play (Schranner et al., 2017; Lynch et al., 2018). The level of heat strain would have been comparable. Additionally, the present environmental conditions do not account for any additional heat load that may arise from direct or indirect solar radiation, which suggests that we need to estimate the effect of solar radiation on heat load in live tennis activities. Thirdly, the participants need to exercise in heat condition, which increase the difficulty of recruiting subjects. Meanwhile, not all participants can complete the exercise protocol. These reasons led to the insufficient number of subjects. We used effect size estimates (Cohen’s d) to assess and categorise efficacy in order to attain more accurate statistics on the small sample. Lastly, we only recruited male participants. The extension of these research questions to the female population would promote the significance and impact of the investigation in the future.

## 5 Conclusion

In a hot/wet environment, pre-cooling and intermittent mixed-cooling strategies can significantly improve exercise time and the executive function of tennis players in a simulated tennis match, especially inhibitory control and cognitive flexibility.

## Data Availability

The original contributions presented in the study are included in the article/supplementary material, further inquiries can be directed to the corresponding authors.
